# INCLUSIVE AGING AND DIGITAL PLACE-MAKING IN AGETECH: THE INCLUDEAGE PROJECT

**DOI:** 10.1093/geroni/igad104.1038

**Published:** 2023-12-21

**Authors:** Judith Sixsmith, Mei Fang

**Affiliations:** University of Dundee, Dundee, Scotland, United Kingdom; University of Dundee, Dundee, Scotland, United Kingdom

## Abstract

This presentation will address the challenges of virtual community inclusion for older people, with a focus on seldom-heard and under-served groups, such as those with intellectual and developmental disabilities (IDD) and those who identify as lesbian, gay, bi, trans + (LGBT+). Given that the COVID-19 pandemic has amplified the digital divide, further excluding marginalized social groups, the aim of this ESRC funded IncludeAge Project is to explore how virtual spaces can be made more inclusive to enhance social inclusion and support health equity. The presentation will consist of two key elements. Firstly, current discourses and definitions of ’community’ and ’inclusion’ in digital spheres will be discussed through findings from a scoping review. Secondly, the presentation will introduce new and innovative ways of digital participatory mapping working alongside older people with IDD and those who identify as part of the LGBT+ community using ArcGIS digital storymapping. This approach offers a unique and powerful way of including under-served groups in the design and development of inclusive AgeTech. The presentation will further highlight the challenges and benefits of digital participation in a post-COVID world, particularly for those who experience exclusion. It will demonstrate the importance of involving under-served groups in the design and development of digital spaces to enhance social inclusion and support health equity. To conclude, we will discuss how these new ideas might be used to shape government policy and organizational practices when it comes to the future of inclusive AgeTech research, design, and development with and for older people.

